# PD-1 blockade combined with anlotinib and bicalutamide for metastatic primary cutaneous apocrine carcinoma: a case report and literature review

**DOI:** 10.3389/fimmu.2026.1802283

**Published:** 2026-07-23

**Authors:** Yuqi Jin, Linglin Fu, Xuan Leng, Yinuo Tan

**Affiliations:** 1 Department of Medical Oncology, Key Laboratory of Cancer Prevention and Intervention, Ministry of Education, The Second Affiliated Hospital, Zhejiang University School of Medicine, Hangzhou, China; 2 Zhejiang Provincial Clinical Research Center for Cancer, Hangzhou, China; 3 Cancer Center of Zhejiang University, Hangzhou, China; 4 Center for Medical Research and Innovation in Digestive System Tumors, Ministry of Education, Hangzhou, China; 5 Department of Infectious Diseases, The Second Affiliated Hospital, Zhejiang University School of Medicine, Hangzhou, China

**Keywords:** androgen receptor, anlotinib, cutaneous apocrine adenocarcinoma, primary cutaneous apocrine carcinoma, serplulimab

## Abstract

**Background:**

Primary cutaneous apocrine carcinoma (PCAC) is a rare malignant adnexal tumor with substantial diagnostic complexity and no established systemic therapy standard for advanced disease.Evidence for endocrine therapy, targeted therapy, and immune checkpoint inhibitors is limited to small series and case reports.

**Case presentation:**

A patient presented with a pruritic erythematous plaque on the left chest wall that progressively enlarged over one year. Skin biopsy and subsequent pathology consultation demonstrated invasive adenocarcinoma involving the dermis and subcutaneous tissue. Immunohistochemistry supported PCAC (CK7+, GATA3+, AR strongly positive, and patchy GCDFP-15 positivity), and a left axillary lymph node confirmed metastatic carcinoma. Whole-body ^18^F-FDG PET/CT revealed extensive metastatic disease involving multiple lymph node stations, lung, pleura, and multiple bones, precluding upfront surgery. The patient received systemic therapy with serplulimab (300 mg intravenously every 3 weeks) plus anlotinib (10 mg orally once daily, days 1–14 of a 21-day cycle) and concomitant bicalutamide (50 mg orally once daily). After two cycles, CT imaging showed partial reduction in pulmonary nodules and mediastinal lymphadenopathy, with thinning of the chest wall lesion and resolution of a small pleural effusion. Subsequent follow-up demonstrated continued clinical improvement of the chest wall lesion and sustained radiological disease control, without new rapidly progressive visceral lesions. No clinically significant adverse events or notable laboratory toxicities were observed during treatment and follow-up.

**Conclusion:**

This case illustrates a feasible combination approach for widely metastatic PCAC using PD-1 blockade, anti-angiogenic therapy, and anti-androgen treatment, with clinical and radiographic improvement and favorable tolerability during follow-up. Prospective data are needed to clarify patient selection and define optimal systemic strategies for advanced PCAC.

## Introduction

Primary cutaneous apocrine carcinoma (PCAC), also referred to as cutaneous apocrine adenocarcinoma, is an exceedingly rare malignant adnexal tumor derived from apocrine sweat glands (1). Owing to its histologic overlap and shared immunophenotypic features with metastatic adenocarcinomas—particularly breast carcinoma—PCAC remains diagnostically challenging and typically requires careful clinicopathologic correlation and systematic exclusion of an extracutaneous primary (2). While wide local excision or Mohs micrographic surgery may be curative in localized disease, advanced or metastatic PCAC lacks a standardized systemic treatment strategy, and current evidence is largely confined to case reports and small case series (3).

Molecular and immunophenotypic profiling has increasingly informed individualized management in this setting (4, 5). A subset of PCAC exhibits hormone receptor and androgen receptor (AR) expression, supporting the rationale for endocrine therapy or anti-androgen treatment (6–8). In addition, immune checkpoint blockade has demonstrated activity in occasional reports of metastatic PCAC, highlighting the potential role of PD-1/PD-L1 inhibitors in selected patients (9, 10). Anti-angiogenic multi-target tyrosine kinase inhibitors may further provide a pathway-aligned option when conventional regimens are limited, and combinations of anti-angiogenic agents with PD-1 blockade have a mechanistic rationale based on modulation of the tumor microenvironment (11–14).

Here, we report a patient with widely metastatic PCAC involving lymph nodes, lung, and bone, who achieved clinical and radiographic improvement after combination treatment with the PD-1 antibody serplulimab, the anti-angiogenic tyrosine kinase inhibitor anlotinib, and the anti-androgen bicalutamide (15–17). This case adds to the limited literature on systemic treatment approaches for advanced PCAC and supports further exploration of biomarker-guided combination strategies (18).

## Case presentation

A 77-year-old male presented with a red plaque on the left chest wall that had persisted for over 1 year. The lesion was initially noticed without an obvious trigger and was accompanied by pruritus but no pain. Over time, the plaque gradually enlarged with ill-defined borders.

At baseline, the patient had an ECOG performance status of 1. He reported preserved oral intake and sleep, with no significant weight loss or systemic symptoms. Physical examination was unremarkable except for the left chest wall lesion. Baseline laboratory tests showed preserved hematologic, hepatic, and renal function. Complete blood count showed a white blood cell count of 7.0 × 10^9^/L, hemoglobin 150 g/L, and platelet count 242 × 10^9^/L. Serum biochemical testing showed total protein 72.7 g/L, albumin 40.6 g/L, total bilirubin 10.0 μmol/L, alanine aminotransferase 16 U/L, serum creatinine 88.5 μmol/L, and an estimated glomerular filtration rate of 72.69 mL/min. These findings indicated that the patient had adequate baseline organ function before systemic therapy.

The patient sought medical attention and underwent a skin biopsy at Hangzhou Third Hospital on May 22, 2025.

### Diagnostic assessment

**Figure 1:**
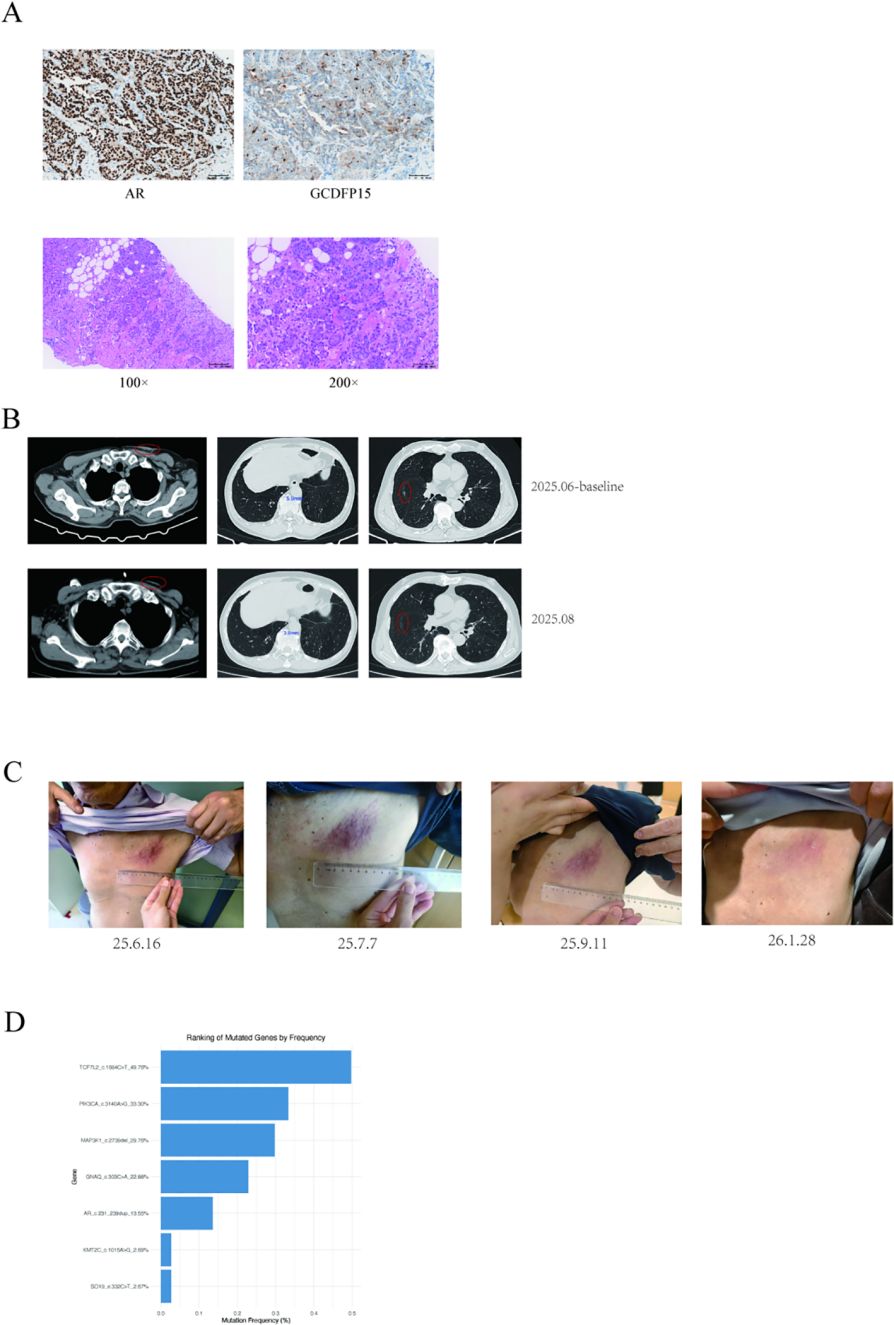
**(A)** Histopathology and immunophenotype of the primary cutaneous lesion (H&E; AR and GCDFP-15 IHC). **(B)** Radiographic response to treatment on chest CT (baseline vs post-treatment). **(C)** Serial clinical photographs showing evolution of the left chest wall plaque during therapy. **(D)** Targeted sequencing results: ranking of mutated genes by frequency in this case.

On June 6, 2025, pathology consultation at our institution reported an invasive adenocarcinoma involving the dermis and subcutaneous tissue, and immunohistochemistry (IHC) showed diffuse positivity for CK7, GATA3, and androgen receptor (AR) with focal/patchy GCDFP-15 positivity (**Figure 1A**), supporting primary cutaneous apocrine carcinoma. In addition, pathology from a left axillary lymph node showed metastatic carcinoma, and in the context of the clinical history, metastatic disease from cutaneous apocrine carcinoma was considered most likely.

Detailed IHC results were as follows: ER 85% (2–3+), PR 80% (2–3+), HER2 (c-erbB-2) 2+ (FISH recommended), Ki-67 20%, AR 90% (3+), GATA3 positive, E-cadherin membranous positive, p120 catenin membranous positive, p63 positive in a small subset of tumor cells, calponin negative, and GCDFP-15 partially positive.

For staging, whole-body ^18^F-FDG PET/CT performed on June 10, 2025 revealed a hypermetabolic nodule in the left chest wall, consistent with malignancy. Multiple sites showed increased FDG uptake suspicious for metastases, including multiple bones (sternum; C7, T1–3, T10, L2 vertebrae and appendages), multiple lymph node stations (bilateral supraclavicular, axillary, mediastinal, and bilateral hilar regions), right pleural small nodules, and thickening of the left chest wall skin with small nodules. Scattered solid nodules in both lungs were also noted, with partial lesions considered metastatic. Importantly, no suspicious breast mass, abnormal breast FDG uptake, or imaging evidence suggestive of a primary breast carcinoma was identified on PET/CT. Additional non-oncologic findings included bronchitis/bronchiolitis changes, emphysema, fibrotic/calcified foci, focal bronchiectasis in lower lobes, and small right pleural effusion.

Based on the extent of systemic disease, surgery was not recommended at that time, and systemic therapy was initiated.

### Therapeutic intervention

The patient received combination therapy consisting of:

Serplulimab 300 mg (intravenous, q3w schedule per cycles documented), plus
Anlotinib hydrochloride 10 mg orally once daily, days 1–14, and
Bicalutamide 50 mg orally once daily.

Documented treatment dates included July 7, 2025, July 28, 2025, and August 18, 2025 for serplulimab plus anlotinib, with continued daily bicalutamide.

### Follow-up and outcomes

A follow-up CT assessment on August 14, 2025, after two cycles of therapy, showed partial reduction in multiple solid pulmonary nodules and mediastinal lymphadenopathy compared with baseline imaging on June 3, 2025. Radiologically, the chest wall/skin lesion also appeared thinner than at baseline, and the previously noted small right pleural effusion had resolved. No clinically significant adverse events were observed, and laboratory examinations did not reveal notable treatment-related toxicity.

The patient subsequently continued combination treatment with serplulimab, anlotinib, and bicalutamide. Another cycle of serplulimab 300 mg plus anlotinib 10 mg once daily on days 1–14 was administered on September 11, 2025, with continued daily bicalutamide. After discharge, the patient remained under regular clinical and radiological follow-up.

During subsequent follow-up, the patient maintained an ECOG performance status of 1, with no clinically significant weight loss, new systemic symptoms, or notable abnormalities on routine laboratory examinations. The left chest wall lesion continued to improve clinically, with gradual flattening of the plaque and fading of erythema. Serial follow-up chest CT examinations demonstrated sustained disease control, with further reduction or stability of pulmonary metastatic lesions and mediastinal lymphadenopathy compared with the initial post-treatment assessment. No new rapidly progressive visceral lesions were identified. The patient tolerated the combined regimen well, without clinically significant immune-related adverse events, severe anti-angiogenic treatment-related toxicities, or notable hepatic, renal, or hematologic abnormalities. The updated serial imaging findings and clinical photographs are shown in **Figures 1B, C**.

## Literature review

To contextualize the present case, we conducted a targeted PubMed literature review focusing on published treatment reports of primary cutaneous apocrine carcinoma/cutaneous apocrine carcinoma. Search terms included “primary cutaneous apocrine carcinoma,” “cutaneous apocrine carcinoma,” “cutaneous apocrine adenocarcinoma,” and “apocrine sweat gland carcinoma,” combined with treatment-related terms including “surgery,” “radiotherapy,” “chemotherapy,” “endocrine therapy,” “anti-androgen therapy,” “HER2,” “targeted therapy,” “immunotherapy,” “PD-1,” and “immune checkpoint inhibitor.” Reports were included in **Table 1** only when the diagnosis was clearly consistent with PCAC/CAC and information on treatment modality and clinical outcome was available.

**Table 1:** Published treatment reports and outcome summary of primary cutaneous apocrine carcinoma (PCAC/CAC).

Author, year	Disease/primary site	Stage/metastasis	Biomarkers	Treatment	Best response/outcome	Follow-up
Singh, 2025 (19)	Primary cutaneous apocrine carcinoma, axilla	Deep axillary lymph node on MRI; one lymph node metastasis confirmed postoperatively	AR/ER/PR/GATA3 positive	Wide local excision + level I/II axillary lymph node dissection + adjuvant radiotherapy + flap reconstruction	No recurrence	6 months
Murayama, 2025 (20)	Metastatic cutaneous apocrine adenocarcinoma	Metastatic disease	NR	Nivolumab	Successfully treated	NR
Ishizuki, 2025 (21)	Metastatic cutaneous apocrine carcinoma	Metastatic disease	NR	Oral S-1 monotherapy	Complete response	NR
Huang, 2024 (22)	Scrotal apocrine carcinoma with Paget change	Postoperative recurrence/metastasis	HER2 status varied between primary and metastatic lesions; metastatic HER2 expression/FISH negative or limited by access	Surgery initially; capecitabine + paclitaxel at recurrence	Promising response	NR
Kamata, 2024 (23)	HER2-positive primary cutaneous apocrine carcinoma, axilla	Axillary lymph node metastases	HER2 positive; ER/PgR positive	Surgery + adjuvant chemotherapy including anti-HER2 agents + hormonal therapy	No recurrence	>3 years
Bouabid, 2024 (24)	Primary cutaneous apocrine carcinoma, thigh	Locally advanced unresectable disease; repeated inguinal lymph nodes/nodules	NR	Definitive radiotherapy, 70 Gy/35 fractions	Disease progression	1 month
Ahmad, 2024 (25)	Apocrine sweat gland carcinoma, axilla	Regional lymph node metastasis after 2 years	NR	Initial complete local excision; surgery + radiotherapy after regional metastasis	No distant disease identified	NR
Tsuruta, 2023 (26)	Cutaneous apocrine carcinoma, single-center retrospective cohort, n = 32	High rate of lymph node/distant metastases at diagnosis; ≥4 positive lymph nodes predicted poor prognosis	NR	Mainly wide local excision + complete lymph node dissection; some patients received adjuvant radiotherapy	Median overall survival 39 months; adjuvant radiotherapy was not associated with significantly different OS	Median OS 39 months
Hidaka, 2012 (27)	HER2-positive metastatic cutaneous apocrine carcinoma	Metastatic disease	HER2 positive	Lapatinib + capecitabine	Successful treatment	NR
Hollowell, 2012 (3)	Cutaneous apocrine adenocarcinoma, SEER cohort, n = 186	Lymph node metastasis common among patients undergoing nodal surgery; lymph node positivity and metastatic disease associated with worse OS	NR	Surgery-based treatment in most patients; node surgery in a subset	Median overall survival 51.5 months; lymph node positivity and metastatic disease associated with worse OS	Median OS 51.5 months
Tlemcani, 2010 (28)	Metastatic apocrine carcinoma, scalp	Metastatic disease	NR	Systemic chemotherapy	Prolonged response	NR
Morabito, 2000 (29)	Recurrent apocrine gland carcinoma, scalp	Recurrent disease after prior surgery/radiotherapy/chemotherapy	NR	Second-line weekly methotrexate + bleomycin	Long-term progression-free survival	NR
Neumann, 1989 (30)	Recurrent apocrine carcinoma, axilla	Multiple local recurrences	NR	Multiple excisions + regional gland removal; tamoxifen failed; local radiotherapy subsequently used	Radiotherapy was effective for local control	NR

## Discussion

Primary cutaneous apocrine carcinoma (PCAC), also termed cutaneous apocrine adenocarcinoma, is an exceptionally rare malignant adnexal tumor arising from apocrine sweat glands (31, 32). Because its histomorphology and immunophenotype can closely resemble metastatic adenocarcinomas—most notably breast carcinoma—diagnosis is often difficult, and, in practice, it hinges on building a coherent clinicopathologic “evidence chain” rather than relying on a single histologic pattern or marker (33, 34). In our case, the diagnostic process was strengthened by integrating routine histology, immunohistochemistry, clinical presentation, metastatic pattern, and systematic imaging evaluation. Importantly, whole-body PET/CT did not reveal any suspicious breast mass, abnormal breast FDG uptake, or imaging evidence suggestive of a primary breast carcinoma. No other extracutaneous primary tumor was identified. These findings, together with the predominant dermal and subcutaneous distribution of the tumor, the compatible apocrine immunophenotype, strong AR expression, patchy GCDFP-15 positivity, and metastatic carcinoma in the ipsilateral axillary lymph node, supported a primary cutaneous origin rather than metastatic breast or visceral adenocarcinoma. Recent clinicopathologic series have shown that many PCACs are consistently positive for CK7, GATA3, and GCDFP-15 and commonly negative for p63, while showing variable expression of EMA/CEA and hormone receptors; importantly, in selected cases, retention of a peripheral myoepithelial layer highlighted by SMA or calponin can support a primary cutaneous adnexal origin, although none of these findings alone is definitive without clinical correlation (35–37). Therefore, comprehensive history-taking and appropriate imaging remain indispensable when PCAC is suspected.

Therapeutically, there is no universally accepted systemic standard for locally advanced or metastatic PCAC, largely because of its rarity, and most evidence is derived from case reports and small series (38, 39). Against this background, we pursued a biologically informed strategy tailored to the tumor’s molecular and immunophenotypic features. First, we selected bicalutamide because an androgen receptor (AR)-driven phenotype has increasingly been recognized in PCAC, and clinical activity of anti-androgen approaches has been reported in metastatic PCAC, including responses to systemic androgen blockade/anti-androgen therapy. Consistent with this rationale, AR positivity was confirmed in our patient’s tumor specimen, which made AR-directed endocrine manipulation a reasonable and comparatively tolerable component of treatment (40).Targeted sequencing further identified recurrent alterations including AR and PIK3CA (**Figure 1D**), which supported the biomarker-informed rationale for combining anti-androgen and pathway-aligned anti-angiogenic strategies.

Anlotinib was incorporated as an anti-angiogenic multi-target tyrosine kinase inhibitor rather than as a PI3K/AKT-targeted agent. Although the patient harbored PIK3CA alterations, these alterations should not be interpreted as predictive biomarkers for anlotinib response. Anlotinib primarily targets VEGFR1–3, FGFR1–3, PDGFR-α, and c-Kit, and its use in this case was based on its anti-angiogenic properties, the lack of established systemic options for metastatic PCAC, and the broader rationale for combining anti-angiogenic therapy with immune checkpoint blockade. Any interaction between angiogenic signaling, PI3K/AKT pathway activity, and immune modulation in this patient remains speculative. Preclinical studies have linked anlotinib and VEGF/VEGFR signaling to PI3K/AKT pathway modulation in other tumor or endothelial models (41–43).

Finally, serplulimab (HLX10), a PD-1 monoclonal antibody, was incorporated because immune checkpoint blockade has shown potential in advanced PCAC and related cutaneous epithelial malignancies, and because durable activity of serplulimab has been demonstrated in previously treated MSI-H/dMMR solid tumors in a phase II setting (44). Although serplulimab-specific evidence in PCAC remains limited, a published case has documented metastatic cutaneous apocrine adenocarcinoma responding to pembrolizumab, suggesting that PD-1 blockade can be effective in at least a subset of PCAC patients (45). Moreover, there is a well-established conceptual basis for combining anti-angiogenic therapy with immune checkpoint inhibitors: VEGF/VEGFR blockade may normalize abnormal tumor vasculature and mitigate VEGF-driven immunosuppression, thereby facilitating immune-cell infiltration and enhancing PD-1/PD-L1 blockade efficacy (46). Taken together, these lines of evidence supported the inclusion of serplulimab as part of our systemic approach (47).

Nevertheless, our report is inherently limited by its single-case design and the absence of standardized comparators. Even so, it underscores a pragmatic direction for advanced PCAC management: when strict diagnostic exclusion of metastasis is achieved, treatment decisions may be rationally aligned with actionable phenotypes such as AR positivity and with pathway-informed strategies such as anti-angiogenic targeting, while PD-1 blockade can be considered in selected scenarios based on emerging evidence and tumor/host context.

## Conclusion

This case describes early radiographic improvement and favorable short-term tolerability in a patient with widely metastatic primary cutaneous apocrine carcinoma treated with combined PD-1 blockade, anti-angiogenic therapy, and anti-androgen treatment. The therapeutic strategy was most directly supported by strong AR expression for anti-androgen therapy and by the general clinical rationale for anti-angiogenic therapy and PD-1 blockade in the absence of established systemic options. In addition, the limited immune biomarker data and short follow-up duration restrict the immunologic and clinical interpretation of this case. Further studies and additional case reports with longer follow-up and comprehensive immune profiling are needed to clarify the role of immunotherapy-based combinations in advanced PCAC.

## Data Availability

The original contributions presented in the study are included in the article/supplementary material. Further inquiries can be directed to the corresponding author.
